# How to Motivate Whole Blood Donors to Become Plasma Donors

**DOI:** 10.1155/2014/752182

**Published:** 2014-10-28

**Authors:** Gaston Godin, Marc Germain

**Affiliations:** ^1^Research Group on Behavior and Health, Laval University, FSI-Vandry, Room 3493, Quebec City, QC, Canada G1V 0A6; ^2^Medical Affairs, Héma-Québec, 1070, avenue des Sciences-de-la-Vie, Québec City, QC, Canada G1V 5C3

## Abstract

This study tested the efficacy of interventions to recruit new plasma donors among whole blood donors. A sample of 924 donors was randomized to one of three conditions: *control; information only by nurse;* and *information plus self-positive image message by nurse* (SPI). Participants in the *control* condition only received a leaflet describing the plasma donation procedure. In the two experimental conditions the leaflet was explained face-to-face by a nurse. The dependent variables were the proportion of new plasma donors and the number of donations at six months. Overall, 141 (15.3%) new plasma donors were recruited at six months. There were higher proportions of new plasma donors in the two experimental conditions compared to the control condition (*P* < .001); the two experimental conditions did not differ. Also, compared to the *control* condition, those in the experimental conditions (all *Ps* < .001) gave plasma more often (*information only by nurse:* *d* = .26; SPI: *d* = .32); the SPI intervention significantly outperformed (*P* < .05) the *information only by nurse* condition. The results suggest that references to feelings of SPI such as feeling good and being proud and that giving plasma is a rewarding personal experience favor a higher frequency of plasma donation.

## 1. Introduction

In the USA, plasma intended for fractionation is obtained mainly from remunerated donors [1]. Therefore, the main challenge for commercial plasma collectors in the US is to apply procedures that will safeguard the quality and safety of the product. In many other countries, however, nonremunerated plasma donation is the usual practice. This means that specific interventions not based on monetary incentives are required to recruit plasma donors.

To our knowledge, in the scientific literature only a few studies have reported the impact of interventions on plasma donor recruitment [2–5]. Each of these interventions mainly provided information about the plasma donation procedure and was successful in recruiting a significant number of new plasma donors. Nonetheless, it was recently shown that one-on-one conversation was the best approach to motivate whole blood donors to become plasma donors especially if the conversation was donor-oriented, that is, focusing on “donor's needs and welfare” [5]. This latter observation is in agreement with the results reported by Ferguson et al. [6], showing that committed blood donors are more willing to donate blood when exposed to a benevolence message. Moreover, their results were consistent with those of Weyant [7] who showed that when helping costs are high (e.g., giving plasma), beliefs in personal benefits are more important for promoting action.

Given the increasing importance for blood agencies to recruit new plasma donors without remuneration, the present research focused on (a) whether two interventions outperformed a* control* condition regarding the recruitment of new plasma donors, (b) whether any of the two interventions engendered a greater number of donations compared to a* control* condition, and (c) the moderating effects of gender, age, and donor status on the findings.

## 2. Methods

### 2.1. Participants and Procedure

**Figure 1:**
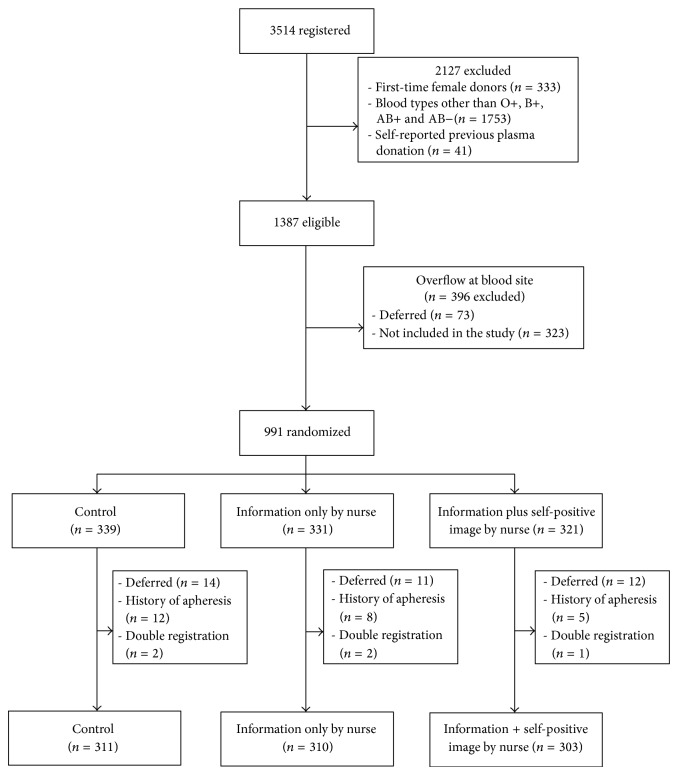


The population targeted by this study was whole blood donors aged 18 to 70 years, who donated at one of the mobile blood drives organized by Héma-Québec, the blood establishment in Quebec. To be included in the study, donors had to be living near the fixed donation center in Quebec City, where apheresis plasma collections are performed. Thus, donors were included if they resided in the metropolitan area or if they gave blood at a mobile clinic within a driving distance of 45 minutes from the apheresis center. A total of 3,514 donors registered at one of the 33 different mobile blood drives held within the specified geographical area between February 14 and June 7, 2012. Donors were excluded if they self-reported plasma donation before (*n* = 41); were first time female donor (*n* = 333); or had blood types other than O+, B+, AB+, and AB− (*n* = 1, 753) (see Figure 1). Thus 1,387 donors were eligible for the study.

Given the variability in size of the blood drives, at each site the number of donors to be recruited was predetermined and varied between 12 and 60. Recruitment of the donors was done at the time of registration, at a pace adapted to the flow of donors, and this is in order to avoid overloading the research nurses. For this latter reason, 396 were not included in the study. The other 991 eligible donors were asked at registration if they agreed to participate in a project aimed at better understanding the motivation of blood donors towards plasma donation. If yes, they were given one of three folders that were placed in a prerandomized order. This was the randomization method applied to allocate blood donors to one of the two interventions or the* control* condition.

Thus, 991 were randomized in one of the three groups. Subsequently, 37 donors were temporarily deferred from giving blood at the time of reviewing their screening questionnaire with the nurse, 25 donors had a history of apheresis according to the donor information system (*Progesa, Mak System*), and five registered twice during the study; thus, these 67 individuals were excluded from the study. In sum, data from 924 blood donors were included in the analyses. The number of blood donors in each condition was 303 (*information only by nurse*); 310 (*information plus self-positive image by nurse*); and 311 (*control*). This study, realized under the legal mandate of Héma-Québec, was conducted to evaluate possible changes in operational recruitment techniques that would have been applied to the donors in any case, except for randomization. Notwithstanding this observation, all standard American Psychological Association (APA) ethical procedures were applied.

### 2.2. Intervention

Participants in the* control* condition were given a folder containing the following: a leaflet explaining the plasma donation procedure (a paragraph of 95 words and a photograph showing someone giving plasma) and a registration form for a first plasma donation. They were only invited to read by themselves these documents. Participants in the* information only by nurse* condition received the same documents as those in the* control* condition but were told that the research nurse would provide further explanations at a later time during the donation process. Thus, upon completion of the donor eligibility questionnaire for blood donation, the trained research nurse briefly reviewed (in less than two minutes) the content of the leaflet describing the plasma donation and the procedure itself, including when, where, and how donors could give plasma. They also answered any questions donors might have in this regard. Participants in the* information plus self-positive image by nurse* condition also reviewed the documents at the time they met the nurse, but the leaflet contained an additional paragraph (101 words) about feelings of self-positive image expressed by plasma donors. For both experimental conditions, five nurses were previously trained to deliver the messages on sites of mobile blood drives and to respond to any questions blood donors might have regarding plasma donation. At random, they met blood donors of both experimental conditions on each site. The review of the leaflet by nurses was done within the usual screening procedure for eligibility to give blood and did not require more than two minutes. Finally, before leaving the site of the blood drive, all donors of the three conditions could insert in an envelope their signed registration form providing consent to be phoned by the staff of the plasma donation center to set up an appointment for a first plasma donation, no earlier than 56 days after their whole blood donation.

### 2.3. Measurement of Donation Behavior

Objective measures of donation behavior were obtained for each participant. Thus, for each blood donor, whether s/he gave a first lifetime plasma donation and the number of plasma donations during the six-month period following the index donation were determined. This information was extracted from the donor information system (*Progesa, Mak System*). An anonymous research code was used to link individuals with their behavioral data.

### 2.4. Overview of Analyses

Data analyses proceeded in five stages. First, demographic and behavioral characteristics of the final sample are described. Second, representativeness and randomization checks are presented. Third, the omnibus tests for the effect of condition (experimental versus* control*) on the proportion of new plasma donors and the mean number of plasma donations over the study period of 6 months. Fourth, planned analyses were undertaken that compared the effect of each intervention on donation behavior, compared with the* control* condition. Analyses were undertaken according to the intention-to-treat analysis. Finally, tests for modification of intervention effects by gender, age, and donor status were conducted via moderated regression analysis.

## 3. Results

### 3.1. Demographic and Behavioral Characteristics of the Sample

The final sample (*N* = 924) consisted of 378 women (40.9%) and 546 men (59.1%). Participants were predominantly repeat donors (87.8%) and had a mean age of 41.5 years (SD = 14.9). Overall, 141 (15.3%) donors gave plasma at least once during the 6-month follow-up period. In total, they made 403 plasma donations.

### 3.2. Representativeness and Randomization Checks

To check on the success of randomization of participants, the three conditions (*control, information only by nurse, and information plus self-positive image by nurse*) were compared on sociodemographic variables (i.e., age, gender, and donor status) at the time of randomization. No difference was observed on any of the variables (gender, *χ* ^2^(2, *N* = 924) = 1.31, *P* = .518; age, *χ* ^2^(4, *N* = 924) = 2.58, *P* = .63; and donor status, *χ* ^2^(2, *N* = 924) = 2.83, *P* = .24), suggesting randomization was successful. In addition, because correlations between these variables and donation (Spearman's coefficient: rho, *ρ*) were low (at 6 months: gender, *ρ* = −.02; age, *ρ* = .06; and donor status, *ρ* = .10), we did not control for these variables in subsequent analyses.

### 3.3. Omnibus Effect of Condition on Plasma Donation Behaviors

The use of a dichotomized measure refers to the number of individuals who gave plasma at least once. The intention-to-treat analysis (GENMOD procedure, Binomial distribution: 1 = gave plasma; 0 = did not give plasma) showed a main effect on the proportion of blood donors in the experimental conditions who gave plasma at least once at 6 months compared to the* control* condition (*χ* ^2^(1, *N* = 924) = 31.97, *P* < .001). For the frequency of plasma donation (GENMOD procedure, Poisson distribution), the analysis showed a main effect for the experimental versus* control* conditions at the 6-month follow-up, (*χ* ^2^(1, *N* = 924) = 5.04, *P* < .05). These findings justify more focused contrasts to assess pairwise differences between conditions.

### 3.4. Pairwise Comparisons of the Impact of Conditions on Plasma Donation Behaviors

Contrast analyses indicated that* information only by nurse* (*χ* ^2^(1) = 25.15, *P* < .001; 19.8%) and* information plus self-positive image by nurse* (*χ* ^2^(1) = 25.01, *P* < .001; 19.7%) showed significant greater proportions of donors who gave plasma at least once compared to* control* (6.4%). The two experimental conditions did not differ (*χ* ^2^(1) = 0.00, *P* = .969).

Table 1 presents the mean frequency of plasma donations for each condition at 6-month follow-up. As expected, the* control* condition exhibited the lowest mean donation (*M* = .135). At the 6-month follow-up, the* information only by nurse* (*χ* ^2^(1, *N* = 924) = 25.22, *P* < .001, *d* = 0.26) and* information plus self-positive image by nurse* conditions (*χ* ^2^(1, *N* = 924) = 52.56, *P* < .001; *d* = 0.32) showed significantly greater frequency of plasma donations compared to the* control* condition. Moreover, the* information plus self-positive image by nurse* significantly outperformed the* information only by nurse *(*χ* ^2^(1, *N* = 924) = 5.04, *P* < .05, *d* = 0.13).

### 3.5. Tests for the Modification of the Intervention Effect on Plasma Donation Behaviors

As recommended by Aiken and West [8], a series of three-step hierarchical regressions were used to test whether gender, age, and donor status moderated the effect of each intervention at the 6-month follow-up. Behavior was regressed on the respective condition at step 1, the three moderator variables were entered on the second step, and the three condition × moderator interaction terms entered the equation on the third step.

Concerning the proportion of whole blood donors who gave plasma at least once, only donor status (*P* < .05) emerged as a moderator for one of the interventions:* information plus self-positive image by nurse*. This latter intervention was significant only among repeat whole blood donors (*B* = 1.36, SE = .28, *P* < .001).

Concerning the mean number of plasma donations, few moderation effects were observed. First, gender, age, and donor status (all *Ps* < .01) moderated the effect of the* information plus self-positive image by nurse* intervention. More precisely, this latter intervention was more efficient among men (*B* = 0.81, SE = .21, *P* < .001), although it remained significant among women (*B* = 1.82, SE = .33, *P* < 001), donors aged 35 year and more (35–49 years: *B* = 1.23, SE = .26, *P* < .001; 50 years and more: *B* = 1.81, SE = .38, *P* < .001), and repeat donors (*B* = 1.24, SE = .18, *P* < .001). Finally, it was observed that age (*P* < .01) moderated the effect of the* information only by nurse* intervention. This intervention was more efficient among donors aged 50 years and more (*B* = 1.78, SE = .38, *P* < .001); it was not significant among the younger groups.

## 4. Discussion

The present study was quite successful and both interventions performed evenly in recruiting new plasma donors although the* information plus self-positive image by nurse* intervention outperformed the* information only by nurse* on the frequency of plasma donations. Three additional observations were noted regarding the moderation effects. First, the effect of both interventions on the frequency of plasma donation was moderated by age, being significant only among older aged groups. Second, the effect of the* information plus self-positive image by nurse* intervention on both outcomes (proportion of new plasma donors and frequency of donation) was moderated by donor status, the effect being significant only among repeat donors. Finally, the* information plus self-positive image by nurse* intervention was more effective to increase the frequency of plasma donation among men, although it was also significant among women.

It is quite interesting to note that only providing basic face-to-face information concerning the plasma donation procedure was sufficient to initiate this new behavior among a substantial proportion of blood donors. This observation runs contrary to the current trend observed in many public health policies to promote the adoption environmental change approaches instead of individuals change strategies. In the present study, our results suggest that providing information is a behavioral change technique that has the potential to change behavior of a significant proportion of blood donors. A number of reasons can explain this positive outcome. First, most blood donors were informed for the first time, face-to-face by a nurse, about the nature of this new behavior and given its similarity with blood donation, they likely evaluated having the capacity to adopt it. Second, the adoption of this new behavior was likely well aligned with the prevailing underlying motivations, for instance to help others. Third, there was a real opportunity to act, with the apheresis center being located within a driving distance of 45 minutes. In summary, in the present case, these above explanations would fit perfectly the COM-B System, a framework for understanding behavior [9]. According to this framework, when individuals have the capacity, are motivated, and have the opportunity to act, there is a high probability that they will take action. Notwithstanding all of these possible explanations, the present study showed that providing face-to-face information changed the behavior of blood donors.

The message referring to self-positive image did not have a significant effect above providing* information only by a nurse* on the recruitment of new plasma donors. However, our self-positive image message resulted in a higher number of donations in the group exposed to the* information plus self-positive image by nurse *intervention compared to* information only by nurse*. This suggests that reference to feelings of self-positive image such as feeling good and being proud and that giving plasma is a rewarding personal experience is a motivational approach to favor a higher frequency of plasma donation. This explanation would be supported by the results of Ferguson and colleagues [6] showing that donors exposed to a benevolent message (i.e., reading a leaflet) highlighting the personally rewarding nature of helping and of self-worth associated with donation significantly increased the willingness to give blood. Our findings are also aligned with the observation reported by Bagot et al. [5] suggesting that messages that emphasize the donor's needs and welfare are more effective to motivate whole blood donors to become plasma donors. Few explanations can be offered to explain this specific finding. First, donors in the SPI condition were described the plasma donation by a nurse as a rewarding experience and told that those who give plasma are proud of doing this, feel good about themselves, and hold personal positive affect (SPI). According to the theory of self-fulfilling prophecy [10], a positive expectation about the adoption of a given behavior (e.g., giving plasma) may affect a person's behavior toward this action in a manner that causes those expectations to be fulfilled. Therefore, once these new plasma donors were convinced that giving plasma really was a rewarding experience, they took very real actions in consequence and made more frequent donations than those who only received information.

Another possible explanation is offered by self-determination theory [11]. This theory would predict that an intrinsic source of motivation better support the adoption and maintenance of a given behavior. Thus, the information provided by nurses can be viewed as a source of external motivation. However, donors in the self-positive image condition may have retained the idea that doing the behavior makes you feel good and, in turn, this makes you do it again. This would then be an internal or intrinsic source of motivation that favors giving plasma again.

The three moderators analyzed, that is, age, gender, and donor status, carried some effect on the effectiveness of our interventions to initiate plasma donation among blood plasma donors. Overall, these moderating effects indicate that interventions to promote starting giving plasma among blood donors are more likely to be successful among a population of men aged 35 years and more and who have some experience with blood donation. There are a number of possible reasons for these moderating effects. For instance, men have a higher circulating blood volume, which enhances the total quantity of plasma that can be collected on the long term compared to women. Individuals aged 35 years and more might also be able to follow a rigorous collection schedule compared to younger individuals [12]. Finally, blood donors who have some experience with the procedure of blood donation likely have a better understanding of the ability required for giving plasma. In summary, this would provide support for policies targeting the recruitment of plasma donors only among those who have given whole blood at least once in their life, such as the case in Australia.

A few limitations of this study should be noted. First, our study was conducted on a sample of French speaking donors in Quebec, Canada, who might differ from other whole blood donors from different countries. Second, five nurses were trained for the present project. It is likely that the quality of the intervention varied from one nurse to another and it is not possible to know if the intervention was always delivered as planned.

## 5. Conclusion

Providing information on how, where, and when to give plasma is enough to convince a significant proportion of whole blood donors to start giving plasma. Moreover, a message highlighting self-positive image of giving plasma appears to enhance the frequency of donation. Blood agencies can therefore introduce this simple low cost promotional approach to recruit new plasma donors, especially among men, aged 35 years and more and who have some experience with giving blood.

## Figures and Tables

**Table 1:** Mean frequency of plasma donations at 6 months (*N* = 924).

Conditions	6 months	
	M	SD
*Information only by nurse *	0.327_b_	0.819
*Information plus self-positive image by nurse *	0.439_c_	1.191
*Control *	0.135_a_	0.648

*Note*. Means within each column that do not share the same subscript differ significantly (*P* < .05, 2-tailed test).
